# Vexilología en el electrocardiograma. Signo de la bandera de Sudáfrica

**DOI:** 10.47487/apcyccv.v3i3.206

**Published:** 2022-09-30

**Authors:** Ruth N. Estupiñan-Paredes, Ana M. Robayo-Betancourt, Nelson L. Moreno-Ruiz

**Affiliations:** 1 Clínica Universitaria Colombia. Bogotá, Colombia. Universidad Nacional de Colombia Clínica Universitaria Colombia Bogotá Colombia

**Keywords:** Infarto de micárdio, Electrocardiograma, Reperfusión Miocárdica, Myocardial Infarction, Electrocardiography, Myocardial Reperfusion

## Abstract

El electrocardiograma (ECG) es un examen que permite tomar decisiones que podrían salvar la vida del paciente. Se debe tener en cuenta que tiene diferentes patrones y diagnósticos diferenciales, como el patrón del síndrome coronario agudo con elevación del segmento ST lateral alto, al cual se denomina el «signo de la bandera de Sudáfrica». Presentamos el caso de un paciente de 44 años que presentó dolor torácico típico en quien se realizó un ECG que evidenció elevación del segmento ST en las derivaciones DI, DII, AVL - V2, e infradesnivel en DIII, lo cual corresponde a una oclusión coronaria aguda con compromiso del segmento lateral del corazón. Este patrón ECG es conocido como el signo de la bandera de Sudáfrica. El pronto reconocimiento permitió la decisión de realizar la terapia farmacológica y la angioplastia de rescate, inmediatamente.

## Abstract (en)

The electrocardiogram (ECG) is a test that will allow making life-saving decisions. It has different patterns, and differential diagnoses, like the pattern of acute coronary syndrome with an elevation of the high lateral ST segment, which is called the sign of the flag of South Africa. We present the case of a 44-year-old patient presented with typical chest pain; the ECG showed ST-segment elevation in the DI, DII, AVL - V2, and ST depression in DIII, corresponding to an acute coronary occlusion with a compromise of the lateral segment of the heart. This ECG pattern is known as the flag sign of South Africa. The early recognition allowed the decision to perform immediately pharmacological reperfusion therapy and rescue angioplasty.

## INTRODUCCIÓN

El electrocardiograma como herramienta accesible y de valor en la identificación diagnóstica y la directriz de las intervenciones de tratamiento en el infarto agudo de miocardio, cobra importancia en los servicios de urgencias. Un análisis detallado del trazado electrocardiográfico que nos permita reconocer precozmente patrones de elevación del segmento ST puede influir en las decisiones sobre el uso de terapia de reperfusión. Es así, que la identificación temprana y precisa de la arteria relacionada con el infarto en el electrocardiograma puede ayudar a guiar intervenciones en los servicios de urgencias ^1^.

Las características electrocardiográficas de los patrones de elevación del segmento ST pueden sesgar las conductas de intervención temprana, como es el caso particular del patrón electrocardiográfico del síndrome coronario agudo con elevación del segmento ST (SCACEST) lateral alto en el que la elevaión de ST se presenta en derivaciones no contiguas, razón por la que se han descrito nemotecnias que facilitan al clínico la asociación de los hallazgos con el diagnóstico electrocardiográfico y, a partir de este, definir intervenciones de manejo tempranas ^2^.

## REPORTE DE CASO

**Figura 1:**
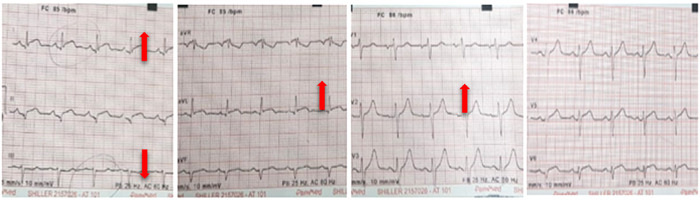
Electrocardiograma al ingreso a emergencia. Las flechas indican los cambios en el segmento ST.

Paciente de 44 años, sin comorbilidades conocidas, ingresa al servicio de urgencias refiriendo cuadro clínico consistente en tres horas de dolor torácico opresivo de intensidad 8/10 en escala análoga del dolor, con irradiación a región interescapular, dolor que se incrementa con el esfuerzo y mejora parcialmente con el reposo, asociado con disnea de pequeños esfuerzos. Inicialmente, realizó una consulta en un centro de atención de primer nivel en Tolima donde se indicó manejo analgésico; sin embargo, el paciente tenía persistencia de dolor, por lo que se le ingresa a un centro de cuarto nivel en Bogotá, en donde le realizan un electrocardiograma (Figura 1). Este examen mostraba supradesnivel del segmento ST en derivaciones DI - DII - aVL - V2, además de infradesnivel en DIII y elevación sutil de segmento ST en v5 y v6, consistente con infarto agudo de miocardio con elevación del segmento ST (IAMCEST) de cara lateral alta.

**Figura 2:**
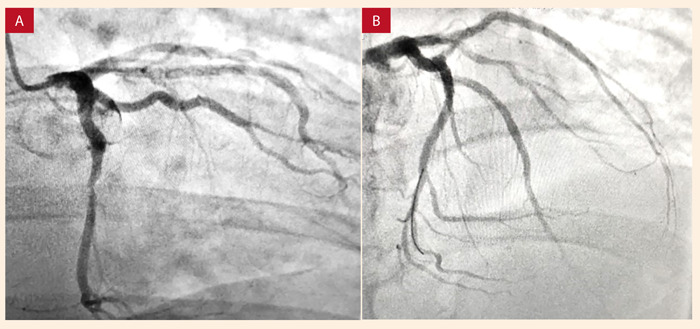
A) Coronariografía donde se evidencia lesión en tercio medio de circunfleja. B) Resultado angiográfico luego de angioplastia e implante de stent.

Se le indicó terapia de reperfusión farmacológica con tenecteplase 50 mg intravenoso con lo que se logró mejoría parcial del dolor sin obtener criterios de reperfusión. Se le indicó angioplastia de rescate, intervención en la que se apreció lesión del 90% en el tercio medio de la arteria circunfleja y oclusión crónica de segunda marginal por lo que se procedió a realizar angioplastia e implante de *stent* medicado en arteria circunfleja, sin complicaciones (Figura 2). Posterior a la intervención, el paciente tuvo una evolución clínica favorable con adecuada tolerancia al tratamiento farmacológico instaurado y sin presentación de síntomas. En el ecocardiograma transtorácico se reportó una fracción de eyección ventricular izquierda (FEVI) del 48%, con alteración de la contractilidad dada por hipoquinesia de segmento basal y medio de pared anterolateral. 

## DISCUSIÓN

En el abordaje inicial del síndrome coronario agudo, la valoración detallada y minuciosa del trazado electrocardiográfico constituye la primera herramienta diagnóstica con implicaciones terapéuticas, que tendrá gran impacto sobre la condición clínica del paciente ^1^. En este escenario clínico, la lectura del electrocardiograma permitirá demostrar la topografía del vaso culpable en los pacientes con síndrome coronario agudo ST elevado, con la sospecha de una oclusión aguda que permita iniciar un tratamiento inmediato ^2^^,^^3^.

**Figura 3:**
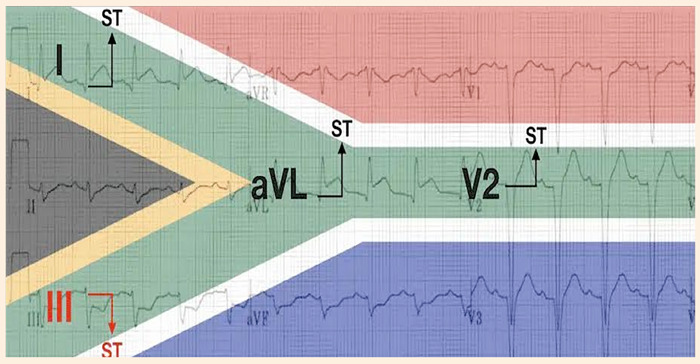
Cambios en el electrocardiograma en el signo de la bandera de Sudáfrica.

El infarto lateral (derivado de la oclusión aguda de la primera rama diagonal de la arteria descendente anterior) con frecuencia, no muestra un patrón de elevación de segmento ST en derivaciones contiguas, lo cual dificulta la aproximación diagnóstica mediante evaluación electrocardiográfica inicial ^2^. En 2016, Littmann propuso una regla mnemotécnica de asociación cuyo objetivo era facilitar la identificación del patrón electrocardiográfico característico en el infarto lateral alto, razón por la que deciden asociar la distribución geométrica, que se marcaba con la combinación de colores en la bandera de Sudáfrica, con las derivadas en las que se presentaban los cambios característicos de la elevación del segmento ST en el infarto lateral alto, denominando esta asociación mnemotécnica como el «signo de la bandera de Sudáfrica» (Figura 3) ^4^. Básicamente, este signo electrocardiográfico se basa en la dirección que adquiere el vector del segmento ST en el IAMCEST lateral alto, el cual se dirige a la axila izquierda entre 0 y -90º en el plano frontal, lo cual da como hallazgos la elevación del segmento ST en las derivaciones I, aVL y V2, con la consiguiente imagen de descenso especular en III y aVF ^5^^,^^6^.

La evidencia con respecto a la aplicación sistemática de este patrón electrocardiográfico es poca, dado que los cambios presentados en la derivación V2 no serían contiguos con los cambios de las derivaciones DI y aVL. Situación que pone de manifiesto el concepto de Littmann en relación a que las derivaciones V1-V2-V3 son también derivadas del plano frontal ^7^^,^^8^. El entender la dirección que adopta el vector de lesión epicárdica hará más sencillo levantar la sospecha del compromiso oclusivo a nivel lateral y guiar la estrategia de reperfusión inmediata ^4^.

Podemos sugerir que este compromiso lateral del corazón, previamente comentado, se verá representado en el estudio de coronariografía por la lesión en el territorio irrigado por la arteria diagonal o circunfleja, de acuerdo con la distribución y tamaño que adopten estas arterias sobre este territorio ^9^.

Si bien el signo de la bandera de Sudáfrica se describió para el compromiso de la arteria primera diagonal, no puede considerarse patognómico de este vaso, dado que en casos de arterias de pequeño calibre (como en el caso del paciente) la irrigación de la porción lateral es más relevante a través del territorio circunflejo, razón por la cual se debe considerar también esta arteria y sus ramas como potenciales generadores de este patrón electrocardiográfico ^10^.

Como regla nemotécnica, la aplicación de la valoración del signo de la bandera de Sudáfrica a la interpretación electrocardiográfica inicial aporta bases de fácil uso y aplicación que facilitan al clínico la identificación temprana de un evento oclusivo agudo del territorio vascular que garantiza intervenciones terapéuticas efectivas.
